# In Memory of Dr. W. Hart Cameron

**Published:** 1886-07

**Authors:** A. G. Rose, C. M. Wright


### OBITUARY.

### In Memory of Dr. W. Hart Cameron.

At a meeting of the dentists of Cincinanti held June 8th, at the Dental College, the following resolution on the recent death of Dr. W. H. Cameron was unanimously adopted:

WHEREAS, by the sudden death by accident of Dr. W. Hart Cameron, eldest son of Dr. J. G. Cameron, and a graduate of the Ohio Dental College, a member of and former officer of the Mississippi Valley Dental Society, a former delegate to the American Dental Association, we the assembled dentists of Cincinnati, recognize the loss of a friend and of a promising young member of the profession, therefore,

*Resolved*, That we tender our heartfelt sympathy to the bereaved family of the deceased; that copies of this resolution be sent to the daily city papers, and to the dental journals of Ohio.

(Signed)

FRANK BELL,

F. A. HUNTER,

H. A. SMITH,

A. BERRY,

F. W. SAGE,

M. A. FLETCHER.

A. G. ROSE, President.
C. M. WRIGHT, Secretary.

